# P-1265. Risk Factors for Contamination of Healthcare Personnel Gloves and Gown with *Candida auris* (*C. auris*)

**DOI:** 10.1093/ofid/ofae631.1447

**Published:** 2025-01-29

**Authors:** Anthony Harris, Sarah E Sansom, Mary K Hayden, David P Calfee, Susan Huang, Mary Bahr-Robertson, Michelle Newman, Raveena D Singh, Raheeb Saavedra, unwana O umana, Lyndsay M O’Hara

**Affiliations:** University of Maryland School of Medicine, Baltimore, Maryland; Rush University Medical Center, Chicago, IL; Rush University Medical Center, Chicago, IL; Weill Cornell Medicine, New York, New York; University of California, Irvine School of Medicine, Irvine, California; University of Maryland Baltimore, Baltimore, Maryland; University of Maryland Baltimore, Baltimore, Maryland; University of California, Irvine School of Medicine, Division of Infectious Diseases, Irvine, California; University of California, Irvine School of Medicine, Division of Infectious Diseases, Irvine, California; Weil Cornell Medicine, New York, New York; University of Maryland School of Medicine, Baltimore, Maryland

## Abstract

**Background:**

*C. auris* is a rapidly emerging pathogen with the ability to spread quickly in healthcare settings. This study aims to describe patient, healthcare personnel (HCP), and environmental risk factors for hospital transmission of *C. auris*.

**Figure:**
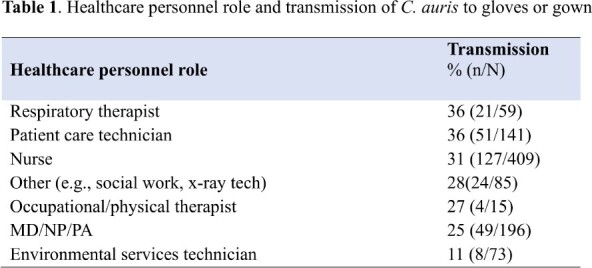

**Methods:**

Patients with a history of *C. auris* were enrolled at 4 hospitals in the US. Upon enrollment, cultures were obtained from patients’ axilla, groin, finger webs, nares, and perirectal area/stool and from 8 environmental surfaces. The research team observed 10 HCP in each patient room and recorded care tasks and all items touched using a standardized data collection form. Prior to room exit, HCP gloves and gown were cultured. The primary outcome was a surrogate measure for transmission defined as HCP glove and/or gown contamination with *C. auris*.

**Figure:**
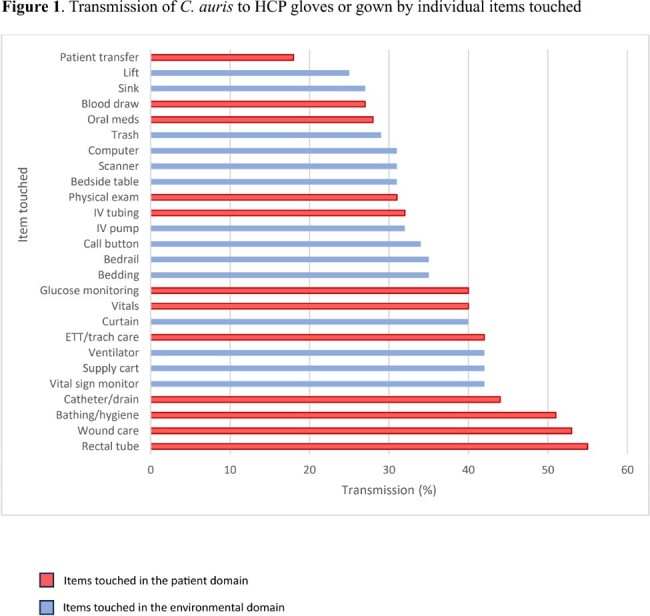

**Results:**

123 patients were enrolled. 25 patients were negative for *C. auris* at all body sites upon enrollment and were removed from analyses. Among the 98 colonized patients, transmission to gloves or gown occurred in 285 (29%) observations. 250 (26%) and 118 (12%) of 980 observations resulted in glove and gown contamination, respectively. Gloves and/or gowns of respiratory therapists (36%) and patient care technicians (36%) were most frequently contaminated (Table 1). Care activities with the highest risk of transmission included handling the rectal tube (55%) and providing wound care (53%). Environmental surfaces with the highest risk of transmission were the vital sign monitor, supply cart, and ventilator (42% each) (Fig. 1). Any interactions that involved touching the patient resulted in glove and/or gown contamination in 252 of /747 (34%) of observations, while touching only the environment resulted in contamination in 33/233 (14%) of observations. Environmental surfaces were frequently contaminated (Fig. 2). Most patients were colonized at multiple body sites (Table 2).

**Figure:**
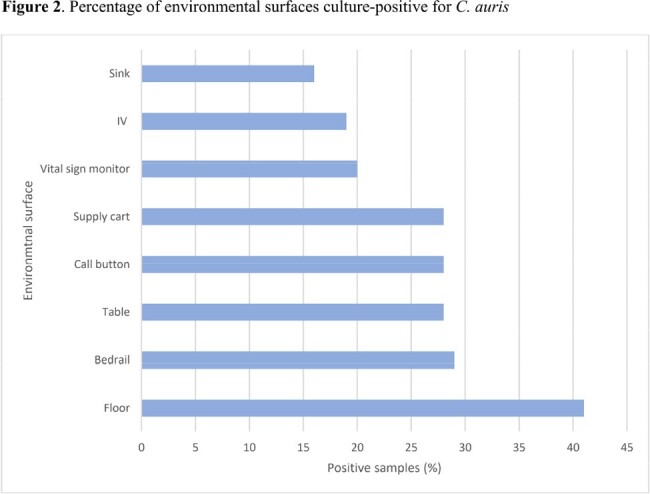

**Conclusion:**

HCP gloves and gowns frequently become contaminated during the routine care of patients with *C. auris*. Extensive colonization at multiple body sites and shed onto environmental surfaces likely contribute to transmission to HCP. These findings highlight the need for additional study of strategies to interrupt transmission and decrease *C. auris*-associated morbidity and mortality.

**Figure:**
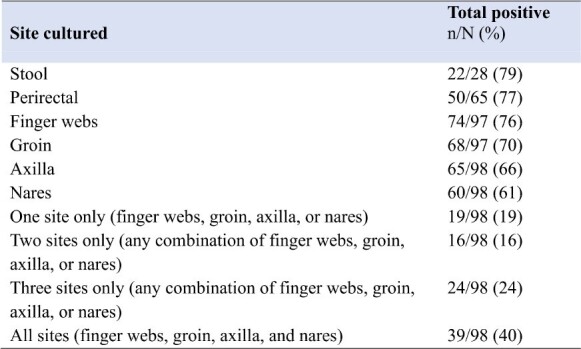

**Disclosures:**

**Anthony Harris, MD, MPH**, Innoviva: Advisor/Consultant|UpToDate: Infection Control Editor **Susan Huang, MD, MPH**, Xttrium Laboratories: Conducting studies in which participating hospital patients received contributed antiseptic products outside the submitted work **Raveena D. Singh, MA**, Xttrium Laboratories: Conducting studies in which participating hospital patients received contributed antiseptic products outside the submitted work **Raheeb Saavedra, AS**, Xttrium Laboratories: Conducting studies in which participating hospital patients received contributed antiseptic products outside the submitted work

